# Metabolic and Molecular Mechanisms of Gemcitabine Resistance in Urothelial Carcinoma

**DOI:** 10.3390/cancers18132126

**Published:** 2026-06-30

**Authors:** Takahisa Yamashita, Shoichi Nagamoto, Masahiro Arai, Sachi Kitayama, Akihiro Yano, Morihiro Higashi

**Affiliations:** 1 Department of Pathology, Saitama Medical Center, Saitama Medical University, 1981, Kamoda, Kawagoe 350-8550, Saitama, Japan; tyamas@saitama-med.ac.jp; 2 Department of Urology, Saitama Medical Center, Saitama Medical University, 1981, Kamoda, Kawagoe 350-8550, Saitama, Japan

**Keywords:** urothelial carcinoma, gemcitabine resistance, chemotherapy resistance, prodrug metabolism, replication stress, apoptosis, tumor microenvironment, autophagy, mucin

## Abstract

Gemcitabine-based chemotherapy has long served as a standard treatment for urothelial carcinoma (UC), particularly in perioperative and metastatic settings. However, therapeutic efficacy is frequently limited by intrinsic or acquired resistance. Gemcitabine functions as a prodrug whose activity depends on coordinated processes involving cellular uptake, intracellular activation, metabolic inactivation, and nucleotide metabolism. Increasing evidence suggests that resistance in UC is mediated by multiple interconnected mechanisms beyond alterations in gemcitabine transport, activation, and inactivation alone. Key molecular determinants include human equilibrative nucleoside transporter 1 (hENT1), deoxycytidine kinase (dCK), cytidine deaminase (CDA), and ribonucleotide reductase regulatory subunit M1 (RRM1), which is involved in nucleotide pool maintenance and DNA synthesis. In addition, replication stress responses, apoptosis evasion pathways, and tumor microenvironment-associated factors also contribute to gemcitabine resistance. Stress-adaptive pathways involving Y-box binding protein 1 (YB-1), hypoxia-inducible factor-1 alpha (HIF-1α), and autophagy-related mechanisms may further promote survival under chemotherapy-induced stress conditions. In addition, extracellular mucin-associated mechanisms may alter intratumoral drug accessibility and contribute to resistance. In this review, we summarize UC-specific evidence regarding gemcitabine resistance and discuss how these pathways collectively shape an integrated resistant phenotype.

## Simple Summary

Gemcitabine-based chemotherapy has long served as a standard treatment for urothelial carcinoma (UC). However, resistance to gemcitabine commonly develops in UC. Although several mechanisms of resistance have been identified in other cancers, those specific to UC have not been comprehensively summarized. This review discusses how altered drug metabolism, cellular stress responses, apoptosis regulation, and tumor microenvironmental factors collectively mediate gemcitabine resistance in UC. Rather than acting independently, these mechanisms appear to form an interconnected network that shapes treatment response. Understanding these pathways may improve future strategies for predicting treatment response and identifying therapeutic targets.

## 1. Introduction

Urothelial carcinoma (UC), arising predominantly in the bladder as well as the renal pelvis and ureter, is one of the most common malignancies of the urinary tract. In advanced disease, systemic chemotherapy has long been the standard treatment. Among chemotherapy regimens, gemcitabine combined with cisplatin (GC therapy) has been widely used as a standard treatment for muscle-invasive bladder cancer before or after cystectomy and for metastatic UC [1]. However, the clinical efficacy of GC therapy remains limited, with overall response rates of approximately 40–50%, while complete responses are observed only in some patients [1,2,3]. Moreover, even tumors that initially respond frequently acquire resistance and eventually relapse, indicating that gemcitabine resistance represents a major clinical challenge in UC management.

In recent years, the therapeutic landscape of UC has rapidly expanded with the introduction of immune checkpoint inhibitors and antibody–drug conjugates [3,4,5,6,7]. Despite these advances, gemcitabine remains clinically relevant because it continues to be used across multiple disease settings, including perioperative chemotherapy for muscle-invasive disease and systemic therapy for advanced or metastatic UC. In addition, gemcitabine-based chemotherapy often serves as an important therapeutic backbone before the introduction of later-line systemic therapies, and prior response to gemcitabine-based chemotherapy and tumor molecular characteristics may influence subsequent treatment selection and overall disease control [8]. Accordingly, understanding the molecular basis of gemcitabine resistance is important not only for optimizing cytotoxic chemotherapy itself but also for improving the effectiveness of subsequent therapeutic strategies in UC.

Gemcitabine (2′,2′-difluorodeoxycytidine) is a nucleoside analog widely used for the treatment of UC. Its antitumor activity depends on a coordinated sequence of events, including cellular uptake by nucleoside transporters, intracellular activation by deoxycytidine kinase (dCK), limited inactivation by cytidine deaminase (CDA), inhibition of ribonucleotide reductase, incorporation into DNA, and induction of replication stress. Disruption of any of these steps can reduce the accumulation or activity of cytotoxic metabolites and contribute to treatment failure. Gemcitabine responsiveness is influenced by multiple molecular mechanisms, including metabolic regulation, intracellular stress responses, and interactions with the tumor microenvironment (TME) [9,10,11,12,13,14,15]. Although many molecular mechanisms associated with gemcitabine resistance have been investigated in pancreatic cancer and non-small-cell lung cancer, several studies suggest that similar pathways may also contribute to therapeutic resistance in UC. However, these mechanisms have not been systematically summarized in the context of UC. A focused UC review is warranted because gemcitabine is used in distinct clinical settings in UC, and resistance mechanisms must be interpreted in relation to urothelial tumor biology, cisplatin-based combination therapy, specimen type, and the tumor microenvironment. This UC-specific perspective is important for translating resistance mechanisms into clinically useful strategies, including biomarker development and therapeutic targeting. A molecule identified in a resistant cell line may provide mechanistic insight, but its clinical value depends on whether it remains predictive in patient specimens obtained before treatment, during therapy, or at recurrence.

In this review, we summarize the molecular mechanisms underlying gemcitabine resistance in UC, focusing on the metabolic network associated with its prodrug nature, intracellular stress response pathways, and TME-mediated extrinsic regulation. We further discuss how these interconnected mechanisms collectively shape the resistant phenotype in UC.

## 2. Molecular Basis of Gemcitabine Resistance: Regulation by the Prodrug Metabolic Network

**Figure 1:**
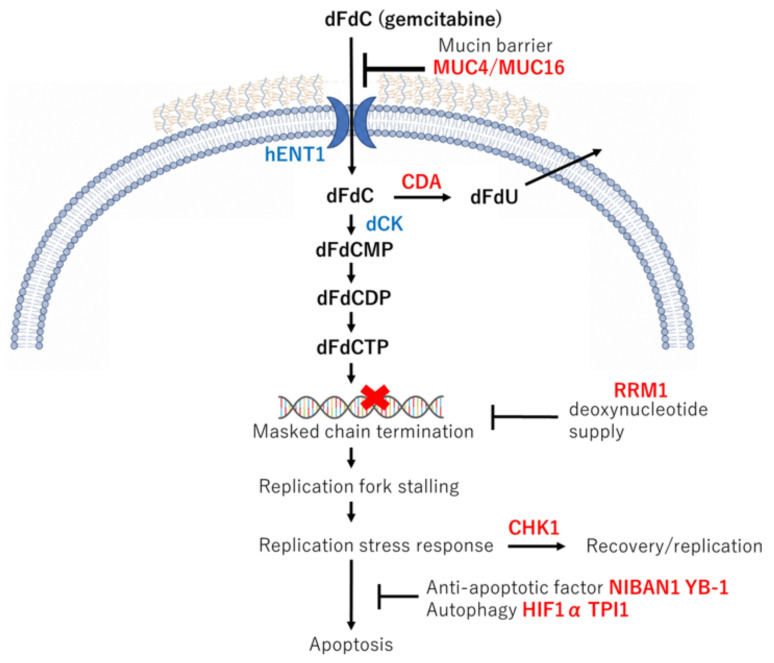
Schematic overview of molecular mechanisms underlying gemcitabine resistance in urothelial carcinoma. Gemcitabine (dFdC) enters tumor cells through the nucleoside transporter, human equilibrative nucleoside transporter 1 (hENT1), whereas mucin-related molecules such as mucin 16 (MUC16) and mucin 4 (MUC4) may interfere with drug uptake and alter the tumor microenvironment. Intracellularly, gemcitabine is activated through sequential phosphorylation by deoxycytidine kinase (dCK), generating active metabolites including gemcitabine diphosphate (dFdCDP) and gemcitabine triphosphate (dFdCTP). Cytidine deaminase (CDA) converts gemcitabine into the inactive metabolite 2′,2′-difluorodeoxyuridine (dFdU), thereby reducing intracellular drug availability. The active metabolite dFdCTP is incorporated into DNA and induces masked chain termination, leading to replication stress. Increased expression of ribonucleotide reductase regulatory subunit M1 (RRM1) counteracts nucleotide depletion and attenuates gemcitabine activity. Replication stress activates checkpoint pathways including checkpoint kinase 1 (CHK1), which promotes replication fork stabilization and cellular adaptation. In contrast, stress-responsive survival factors such as Y-box binding protein 1 (YB-1), Niban apoptosis regulator 1 (NIBAN1), hypoxia-inducible factor-1 alpha (HIF-1α), and triosephosphate isomerase 1 (TPI1) contribute to apoptosis evasion through adaptive signaling pathways including autophagy, metabolic reprogramming and multidrug resistance-associated signaling. These interconnected mechanisms, which have been reported in UC or bladder cancer models, may collectively contribute to the acquisition of gemcitabine resistance. The strength of evidence differs among individual pathways, and mechanisms with limited direct evidence in UC are specifically discussed in the text. Molecules shown in blue indicate factors whose reduced expression or activity contributes to gemcitabine resistance, whereas molecules shown in red indicate factors whose increased expression or activation promotes resistance.

The antitumor activity of gemcitabine is regulated by a coordinated metabolic network involving cellular uptake, intracellular activation, drug inactivation, and nucleotide metabolism. Because gemcitabine functions as a prodrug, alterations in any of these sequential processes can substantially influence drug sensitivity and contribute to the development of resistance. Thus, gemcitabine resistance should not be regarded as the consequence of a single molecular abnormality, but rather as the result of dysregulation across an interconnected metabolic network. In UC, multiple molecules involved in these pathways have been implicated in therapeutic responsiveness. In this section, we summarize the major metabolic regulators associated with gemcitabine resistance in UC (Figure 1).

### 2.1. Human Equilibrative Nucleoside Transporter 1 (hENT1)

The cellular uptake of gemcitabine primarily depends on human equilibrative nucleoside transporter 1 (hENT1), and reduced hENT1 expression represents an important mechanism limiting intracellular drug accumulation [11]. Because gemcitabine is hydrophilic and cannot efficiently diffuse across the plasma membrane, transporter-mediated uptake is a prerequisite for intracellular activation. Therefore, insufficient hENT1 expression or impaired transporter function can reduce the amount of gemcitabine available for phosphorylation by dCK and subsequently decrease the generation of active cytotoxic metabolites. The association between hENT1 expression and treatment responsiveness has also been reported in UC, suggesting that hENT1 is a key determinant of gemcitabine sensitivity [16,17,18,19]. From a clinical perspective, hENT1 has attracted attention as a potential predictive biomarker for gemcitabine-based therapy. However, its clinical utility may depend on the treatment context, assay platform, cut-off value, and whether hENT1 expression reflects functional membrane-localized transporter activity. Thus, hENT1 should be interpreted not only as an expression marker but also as part of the broader pharmacologic pathway that determines intracellular gemcitabine exposure. In addition to expression levels, hENT1 function is also regulated by post-translational modifications. In UC cells, alterations in N-glycosylation have been reported to affect membrane localization and transport activity of hENT1. Reduced β1,6-branched N-glycan modification mediated by N-acetylglucosaminyltransferase V (GnT-V) decreases membrane localization of hENT1, thereby limiting gemcitabine uptake and contributing to its resistance [20]. These findings suggest that resistance may arise not only from quantitative downregulation of hENT1 but also from qualitative impairment of transporter localization or function, an important consideration for biomarker development because total hENT1 expression may not always reflect functional transporter activity at the cell membrane.

### 2.2. Deoxycytidine Kinase (dCK)

Activation of gemcitabine is initiated by intracellular deoxycytidine kinase (dCK). dCK catalyzes the initial phosphorylation of gemcitabine to gemcitabine monophosphate (dFdCMP), which is subsequently converted to gemcitabine diphosphate (dFdCDP) and ultimately the active metabolite gemcitabine triphosphate (dFdCTP) [11,21,22]. Incorporation of dFdCTP into DNA induces “masked chain termination,” in which one additional nucleotide is incorporated after gemcitabine insertion before DNA synthesis is halted [11,21,22]. In parallel, dFdCDP inhibits ribonucleotide reductase (RNR), resulting in depletion of intracellular deoxynucleotide pools [11,21,22]. Through these dual mechanisms, gemcitabine potently suppresses DNA synthesis.

In UC, impaired intracellular activation of gemcitabine represents an important mechanism of chemoresistance. As the rate-limiting enzyme in gemcitabine metabolism, dCK determines whether intracellularly transported drug can be converted into cytotoxic metabolites. Reduced dCK expression or activity limits production of cytotoxic metabolites, thereby attenuating antitumor efficacy. In UC cell lines, reduced dCK expression has been directly associated with gemcitabine resistance, whereas restoration of dCK expression resensitized resistant cells to gemcitabine treatment [23]. Furthermore, variability in dCK expression among UC tumors may partly contribute to the heterogeneous sensitivity observed in gemcitabine-based chemotherapy. However, the clinical utility of dCK as a predictive biomarker in UC remains controversial. In patients with muscle-invasive bladder cancer treated with platinum/gemcitabine neoadjuvant chemotherapy, dCK expression in pretreatment transurethral resection of bladder tumor (TURBT) specimens did not predict pathological complete response [17]. This discrepancy may reflect differences between experimental and clinical settings. Cell-line studies assess tumor cell-intrinsic sensitivity to gemcitabine, whereas clinical response in MIBC is usually evaluated after gemcitabine–cisplatin combination chemotherapy and may be influenced by cisplatin sensitivity, intratumoral heterogeneity, and the tumor microenvironment. Therefore, dCK may be mechanistically important for gemcitabine activation, but its value as a standalone clinical biomarker may be limited without integration with other treatment-response determinants.

### 2.3. Cytidine Deaminase (CDA)

Cytidine deaminase (CDA) plays a central role in gemcitabine inactivation. CDA converts gemcitabine into 2′,2′-difluorodeoxyuridine (dFdU) through deamination, thereby reducing the intracellular availability of active metabolites [11,21]. Increased CDA activity therefore represents an important mechanism of gemcitabine resistance. In contrast to hENT1 and dCK, which regulate drug entry and activation, CDA acts as a negative regulator of gemcitabine exposure by accelerating metabolic inactivation. Thus, the balance between dCK-mediated activation and CDA-mediated inactivation is likely to influence the amount of active gemcitabine metabolites retained within tumor cells. In UC cells, HYAL4-V1-driven CD44-JAK2/STAT3 signaling upregulated CDA expression, resulting in suppression of gemcitabine activity and increased efflux of dFdU [24]. Importantly, CDA inhibition restored gemcitabine sensitivity in resistant tumors, suggesting that CDA-dependent metabolic inactivation may represent a therapeutically targetable resistance mechanism in UC. This finding is clinically relevant because CDA is not only a candidate resistance biomarker but also a potentially actionable enzyme. Pharmacologic inhibition of CDA or strategies that bypass CDA-dependent inactivation may therefore represent rational approaches for restoring gemcitabine sensitivity, although clinical validation in UC remains necessary. Furthermore, CDA expression is influenced by inflammatory signaling and TME. Tumor-associated macrophages (TAMs), which exhibit high CDA activity, can metabolize gemcitabine within TME and thereby reduce local drug availability, promoting chemoresistance [25]. However, although similar mechanisms may also operate in UC, direct evidence linking TAM-associated CDA activity to gemcitabine resistance in UC has not been well established. Therefore, TAM-associated CDA activity should currently be interpreted as a microenvironment-related and hypothesis-generating mechanism in UC rather than as a fully established UC-specific resistance pathway. This distinction is important because tumor cell-intrinsic CDA upregulation has direct experimental support in UC, whereas the contribution of stromal or immune cell-derived CDA to gemcitabine resistance in UC requires further investigation.

### 2.4. Ribonucleotide Reductase Regulatory Subunit M1 (RRM1)

Ribonucleotide reductase regulatory subunit M1 (RRM1) is a catalytic subunit of RNR, which maintains intracellular deoxynucleotide pools required for DNA synthesis. The gemcitabine metabolite dFdCDP inhibits RNR, thereby depleting intracellular deoxynucleotide pools and suppressing DNA synthesis [11,21]. Thus, RRM1 is distinct from classical drug transporters or drug-metabolizing enzymes; it functions mainly in nucleotide pool maintenance and DNA synthesis dynamics, which are directly linked to gemcitabine activity. Increased RRM1 expression counteracts this effect by maintaining deoxynucleotide supply and attenuating gemcitabine-induced inhibition of DNA synthesis. In this context, RRM1 overexpression can reduce the pharmacologic impact of dFdCDP even when gemcitabine uptake and activation remain intact. This mechanism may allow tumor cells to sustain DNA synthesis under gemcitabine-induced metabolic stress and thereby contribute to treatment resistance. In UC, elevated RRM1 expression has been associated with reduced sensitivity to gemcitabine-based therapy and unfavorable clinical outcomes [26,27,28,29]. In vitro studies further demonstrated that RRM1 knockdown enhanced gemcitabine sensitivity and suppressed tumor growth in xenograft bladder cancer models, whereas RRM1 overexpression reduced gemcitabine responsiveness [30,31]. Together, these clinical and experimental findings support RRM1 as a clinically relevant predictive biomarker candidate and a potential therapeutic target in gemcitabine-based treatment for UC.

### 2.5. Replication Stress Response

Gemcitabine impairs replication fork progression during S phase, leading to replication fork stalling and induction of replication stress. Accumulation of single-stranded DNA activates the ataxia telangiectasia and Rad3-related (ATR)–checkpoint kinase 1 (CHK1) pathway, resulting in intra-S-phase checkpoint activation and stabilization of stalled replication forks [32]. This checkpoint response temporarily protects cells by preventing premature cell-cycle progression and allowing time for recovery of stalled replication forks under stress conditions. Accordingly, replication arrest does not necessarily result in immediate cell death. If stalled replication forks are successfully stabilized and restarted, cells may survive despite gemcitabine exposure. In contrast, failure of fork protection results in replication fork collapse, accumulation of DNA double-strand breaks, and subsequent induction of apoptosis. Thus, the biological consequence of gemcitabine-induced replication stress depends not only on the extent of DNA synthesis inhibition but also on the capacity of tumor cells to activate checkpoint-mediated adaptation. In this context, CHK1 functions as a central survival node that links replication stress sensing to cell-cycle control and DNA damage tolerance. In UC, CHK1 inhibition enhanced DNA damage accumulation and promoted apoptosis following gemcitabine exposure, suggesting that CHK1-mediated checkpoint adaptation plays an important role in survival of UC cells under gemcitabine-induced replication stress [33]. These findings suggest that CHK1 inhibition may enhance gemcitabine sensitivity in UC models. Although gemcitabine combined with ATR–CHK1 pathway inhibition has been explored clinically in other solid tumors, clinical validation of this strategy in UC remains limited.

### 2.6. Apoptosis Regulation

Evasion of apoptosis represents an important mechanism underlying gemcitabine resistance, as tumor cells can survive despite persistent replication stress and DNA damage induced by chemotherapy. Several stress-adaptive pathways have been implicated in suppression of apoptosis in gemcitabine-resistant UC. This process is closely linked to the replication stress response described above. When gemcitabine-induced DNA damage is insufficient to trigger irreversible cell death, tumor cells may enter a state of stress adaptation in which checkpoint signaling, survival kinase pathways, autophagy, and metabolic remodeling collectively promote survival. Therefore, apoptosis evasion should be considered not only a downstream consequence of reduced drug cytotoxicity but also an active resistance program that allows tumor cells to tolerate gemcitabine-induced stress. The Niban apoptosis regulator 1 (NIBAN1) has emerged as a potential mediator of gemcitabine resistance in bladder cancer [34]. Proteomic analysis of recurrent NMIBC following intravesical gemcitabine treatment identified NIBAN1 as a highly upregulated molecule associated with gemcitabine resistance. Mechanistically, NIBAN1 activated FAK/SRC/AKT signaling and suppressed caspase-3 activity, thereby promoting cell survival and attenuating chemotherapy-induced apoptosis. These findings suggest that NIBAN1 may function as a bridge between extracellular stress signals and intracellular anti-apoptotic signaling, making it a potential biomarker of survival pathway activation in gemcitabine-resistant tumors.

In addition to direct suppression of apoptotic pathways, stress-responsive survival mechanisms may further enhance resistance to chemotherapy. The stress-responsive transcription factor Y-box binding protein 1 (YB-1) has emerged as a mediator of chemoresistance. In response to cellular stress, YB-1 translocates into the nucleus and regulates transcriptional programs involved in cell survival, stress adaptation, and multidrug resistance-associated pathways, thereby facilitating evasion of apoptosis. In UC, nuclear localization of YB-1 has also been associated with gemcitabine resistance [35]. Because YB-1 can coordinate multiple stress-response genes, its nuclear localization may reflect a broader adaptive phenotype rather than a single downstream event. This feature may partly explain why YB-1 is associated with resistance to different classes of anticancer agents. Additionally, hypoxia reduced the sensitivity to gemcitabine through HIF-1α-associated signaling pathways in UC [36,37]. Hypoxia-induced autophagy suppressed chemotherapy-induced apoptosis, whereas inhibition of autophagy or HIF-1α restored chemosensitivity. Similar autophagy-mediated survival mechanisms have also been implicated in gemcitabine-resistant UC. Triosephosphate isomerase 1 (TPI1), a glycolytic enzyme that has also been implicated in regulation of autophagy-related survival pathways, was found to be upregulated in gemcitabine-resistant bladder cancer tissues and cell lines [38]. TPI1-mediated autophagy and mitophagy protected tumor cells from gemcitabine-induced cell death, thereby enhancing chemoresistance. These observations highlight the close relationship between hypoxia, metabolic adaptation, autophagy, and apoptosis suppression in gemcitabine-resistant UC. In particular, autophagy may act as a protective mechanism by removing damaged organelles and maintaining cellular energy balance under chemotherapy-induced stress. This concept is clinically relevant because targeting only one survival pathway may be insufficient when parallel adaptive mechanisms are active. Collectively, these findings suggest that stress-adaptive survival pathways and autophagy-mediated suppression of apoptosis contribute to gemcitabine resistance in UC.

## 3. Role of Extracellular Mucin in Gemcitabine Resistance

Tumor microenvironmental factors may also contribute to gemcitabine resistance in UC [39]. However, compared with pancreatic cancer, where tumor microenvironment-mediated resistance mechanisms have been extensively investigated, direct evidence in UC remains relatively limited. Unlike tumor cell-intrinsic mechanisms such as altered drug uptake, activation, or DNA damage response, TME-mediated resistance may operate by modifying drug delivery, extracellular drug retention, stromal architecture, and interactions between tumor cells and surrounding immune or stromal cells. Therefore, these mechanisms may reduce effective drug exposure even when tumor cells retain intrinsic sensitivity to gemcitabine. Mucin accumulation within tumor tissues may influence the intratumoral distribution and diffusion of anticancer drugs [40]. In particular, mucin-related molecules such as MUC4 and MUC16 (CA125) have been associated with gemcitabine resistance in bladder cancer [41,42,43]. Proposed mechanisms underlying MUC4- and MUC16-mediated gemcitabine resistance include the formation of a physical mucin barrier, electrostatic inhibition of drug diffusion, and the short half-life of gemcitabine itself. MUC16-positive bladder cancers have also been reported to exhibit an immunosuppressive tumor microenvironment, although direct evidence linking immune suppression to gemcitabine resistance in UC remains limited. Thus, mucin-related molecules may represent not only physical barriers to drug penetration but also indicators of broader microenvironmental remodeling. Further studies are needed to determine whether mucin expression can be used as a predictive biomarker for gemcitabine response or as a target to improve drug delivery in UC. Table 1 summarizes major molecules implicated in gemcitabine resistance in UC and their proposed mechanisms.

## 4. Future Directions

Future studies should focus on validating clinically actionable biomarkers and integrating molecular profiling with therapeutic response data to improve patient stratification and identify novel therapeutic targets. Clinically, the molecules reviewed here may serve as candidate predictive biomarkers for identifying patients likely to benefit from gemcitabine-based therapy and as potential therapeutic targets for overcoming acquired resistance. However, most candidates remain insufficiently validated in prospective UC cohorts, and their clinical implementation will require standardized assays and treatment-specific validation. Importantly, biomarker development should be performed in clearly defined clinical settings, because mechanisms associated with failure of intravesical gemcitabine in NMIBC may differ from those associated with resistance to systemic GC therapy in MIBC or metastatic UC. Future studies should therefore incorporate treatment setting, sample timing, and combination agents into biomarker analysis. In particular, paired pretreatment and post-treatment specimens may help distinguish baseline resistance mechanisms from adaptive changes acquired under therapeutic pressure. Patient-derived bladder cancer organoid models may provide a useful platform for future gemcitabine resistance research [44,45,46]. These models can preserve key molecular and phenotypic features of the original tumors and enable ex vivo assessment of drug sensitivity, clonal evolution, and treatment-induced adaptation [44,45,46]. Therefore, integrating organoid-based drug testing with molecular profiling may help validate candidate biomarkers and support personalized therapeutic strategies in UC. Organoid-based pharmacotyping may also provide functional evidence that complements static molecular biomarkers. For example, molecular alterations in hENT1, dCK, CDA, RRM1, CHK1, or apoptosis-related pathways could be interpreted together with direct ex vivo drug-response data. Such integrated approaches may help identify whether resistance in an individual tumor is primarily driven by impaired drug accumulation, altered nucleotide metabolism, enhanced replication stress tolerance, apoptosis evasion, or microenvironment-related factors. Although organoid systems do not fully reproduce the immune and stromal components of the native TME, they offer a practical platform for linking molecular profiles to drug sensitivity and for prioritizing candidate therapeutic combinations.

## 5. Conclusions

Gemcitabine resistance in UC is mediated by multiple interconnected mechanisms involving drug transport, metabolic activation and inactivation, DNA damage responses, apoptosis regulation, and tumor cell survival pathways. Because gemcitabine functions as a prodrug, alterations in its metabolic network, including dysregulation of hENT1, dCK, CDA, and RRM1, play central roles in determining therapeutic sensitivity. In parallel, dysregulation of apoptosis and autophagy-associated survival pathways has also been implicated in gemcitabine resistance. Extracellular mucin-associated mechanisms may further modulate intratumoral drug distribution and contribute to resistance. Compared with pancreatic cancer, where the molecular mechanisms of gemcitabine resistance have been extensively investigated, the available evidence in UC remains relatively limited. Many proposed resistance mechanisms in UC are still supported primarily by experimental studies, and clinically validated biomarkers for predicting gemcitabine response are lacking. Collectively, gemcitabine resistance in UC should be viewed as a dynamic network involving metabolic adaptation, stress response pathways, and tumor microenvironmental interactions rather than isolated molecular events. This network-based perspective has important clinical implications. Single molecules such as hENT1, dCK, CDA, or RRM1 may provide useful information regarding specific steps of gemcitabine handling, but they may not fully capture the overall resistant phenotype when downstream stress adaptation, apoptosis evasion, and microenvironmental barriers are simultaneously active. Therefore, future biomarker development should move beyond single-marker evaluation and incorporate integrated models that reflect drug uptake, activation, inactivation, DNA metabolic regulation, replication stress tolerance, and cell survival pathways. Such approaches may improve patient stratification and help identify tumors that are unlikely to respond to gemcitabine-based therapy. In addition, several molecules reviewed here may represent potential therapeutic targets for overcoming acquired resistance. Strategies aimed at restoring intracellular gemcitabine exposure, inhibiting metabolic inactivation, disrupting replication stress adaptation, or suppressing autophagy-mediated survival may enhance gemcitabine sensitivity in selected tumors. However, translating these concepts into clinical practice will require prospective validation in UC-specific cohorts, standardized assays, and functional models that can evaluate drug response in a clinically relevant context. A better understanding of the relative contribution of tumor cell-intrinsic and microenvironment-mediated mechanisms will be essential for designing rational combination strategies and improving the clinical benefit of gemcitabine-based treatment in UC.

## Figures and Tables

**Table 1:** Molecular mechanisms implicated in gemcitabine resistance in urothelial carcinoma.

Category	Molecule	Expression Change Associated with Resistance	Mechanism	Evidence in UC [Ref]
Drug uptake	hENT1	↓	Reduced gemcitabine uptake	Clinical + experimental [16,17,18,19,20]
Drug activation	dCK	↓	Reduced active metabolite formation	Clinical + experimental [17,23]
	CDA	↑	Increased dFdU production	Experimental [24]
DNA metabolism	RRM1	↑	Maintenance of nucleotide pools	Clinical + experimental [26,27,28,29,30,31]
Replication stress response	CHK1	↑	Fork stabilization and checkpoint adaptation	Experimental [33]
Apoptosis regulation	NIBAN1	↑	FAK/SRC/AKT activation and apoptosis suppression	Experimental [34]
	YB-1	↑	Stress adaptation and multidrug resistance signaling	Experimental [35]
	HIF-1α	↑	Hypoxia-induced autophagy	Experimental [36,37]
	TPI1	↑	Autophagy-mediated survival	Experimental [38]
Tumor microenvironment	MUC4/MUC16	↑	Extracellular mucin barrier	Experimental [41,42,43]

CDA: cytidine deaminase, CHK1: checkpoint kinase 1, dCK: deoxycytidine kinase, HIF-1α: hypoxia-inducible factor-1 alpha, hENT1: human equilibrative nucleoside transporter 1, NIBAN1: Niban apoptosis regulator 1, RRM1: ribonucleotide reductase regulatory subunit M1, TPI1: triosephosphate isomerase 1, YB-1: Y-box binding protein 1. ↑ indicates increased expression or activation associated with gemcitabine resistance; ↓ indicates decreased expression or activity associated with gemcitabine resistance.

## Data Availability

No new data were created or analyzed in this study. Data sharing is not applicable to this article.

## Abbreviations

The following abbreviations are used in this manuscript:

ATR	ataxia telangiectasia and Rad3-related
CDA	cytidine deaminase
CHK1	checkpoint kinase 1
dCK	deoxycytidine kinase
GC	gemcitabine/cisplatin
HIF-1α	hypoxia-inducible factor-1 alpha
hENT1	human equilibrative nucleoside transporter 1
NIBAN1	Niban apoptosis regulator 1
NMIBC	non-muscle-invasive bladder cancer
RNR	ribonucleotide reductase
RRM1	ribonucleotide reductase regulatory subunit M1
TAM	tumor-associated macrophage
TME	tumor microenvironment
TPI1	triosephosphate isomerase 1
UC	urothelial carcinoma
YB-1	Y-box binding protein 1
